# Metabolomics analysis reveals distinct profiles of nonmuscle‐invasive and muscle‐invasive bladder cancer

**DOI:** 10.1002/cam4.1109

**Published:** 2017-08-01

**Authors:** Divya Sahu, Yair Lotan, Bryan Wittmann, Bruce Neri, Donna E. Hansel

**Affiliations:** ^1^ Department of Pathology University of California at San Diego La Jolla California; ^2^ Department of Urology University of Texas Southwestern Medical Center Dallas Texas; ^3^ Metabolon Inc. Durham North Carolina

**Keywords:** Gas chromatography, liquid chromatography, mass spectrometry, metabolic networks and pathways, metabolomics, urinary bladder neoplasms, urothelium

## Abstract

Urothelial carcinoma is the most common form of bladder cancer, but pathway changes that occur with stage‐wise progression have not been well defined. We used a metabolomics approach to identify potential metabolic pathways uniquely altered in normal urothelium, nonmuscle‐invasive bladder cancer (NMIBC), and muscle‐invasive bladder cancer (MIBC). We performed global metabolomic profiling using GC‐mass spectrometry (MS) and LC‐MS platforms to identify metabolite signatures between normal urothelium and high‐grade urothelial carcinoma of different stages. Pathways globally dysregulated in cancer relative to normal urothelium included glucose, tricarboxylic acid (TCA) cycle, lipid, amino acid, and nucleotide pathways. Urothelial carcinoma showed elevated glucose utilization for glycolysis and increased sorbitol pathway intermediates, consistent with Warburg effect. Anaplerosis to sustain energy production suggested by increased late TCA cycle intermediates, amino acids, and dipeptides occurs in bladder cancer. Urothelial carcinoma also shows altered membrane lipid membrane metabolism and differential derivation of nucleic acid components pyrimidine and purine. In stage comparison, MIBC appears to preferentially enhance cyclooxygenase (COX) and lipoxygenase (LOX) signaling, increase heme catabolism, and alter nicotinamide adenine dinucleotide (NAD+) synthesis with a possible influence from associated inflammatory cells. We identify numerous metabolomic alterations in NMIBC and MIBC that likely reflect underlying pathway changes. Differential pathway activity may have value in designing stage‐specific novel therapeutics in urothelial carcinoma.

## Introduction

Bladder cancer affects more than 400,000 patients worldwide annually 1, 2. The majority of cases consist of noninvasive low‐grade and high‐grade urothelial carcinoma (Ta, Tis) and early invasive urothelial carcinoma (T1), collectively termed nonmuscle‐invasive bladder cancer (NMIBC). Whereas low‐grade papillary urothelial carcinoma progresses only in a minority of patients, those with high‐grade Ta, Tis, or T1 disease have a markedly increased risk of progression. Patients with NMIBC are treated with transurethral resection (TURBT) and, for high‐grade disease, with intravesical therapy that includes Bacillus Calmette‐Guérin (BCG, modified mycobacterium) 3. Although BCG can be effective to delay or prevent disease progression in a subset of patients, a significant proportion of patients ultimately develop invasive disease. Moreover, given the recent global shortage of BCG, there is a need for alternative, rational therapies in this population. In contrast, the primary treatment for patients with muscle‐invasive bladder cancer (MIBC) includes radical cystectomy and bilateral regional lymph node dissection with or without neoadjuvant chemotherapy or chemoradiation.

One approach to identify differentially activated and potentially actionable pathways in these different patient populations is metabolomic profiling. Metabolomic signatures, which reflect the overall biological activity inherent in each cancer, may be used to detect unique pathways activated in cancer subtypes and has been applied to breast, ovarian, head and neck, colorectal, and hepatocellular carcinomas 4. Prior studies using metabolomic analysis of bladder cancer have focused primarily on screening and detection applications using urine 5, serum 6, and cell culture 7. Several additional studies have identified differences between normal urothelium and neoplastic bladder tissue and have identified a subset of metabolites that appear to be unique in these populations 4, 8, 9.

In our study, we have used a unique and highly sensitive approach to expand the identification of altered metabolites in bladder cancer. We have also included high‐grade urothelial carcinomas of different stages to identify unique signatures that distinguish NMIBC from MIBC, given that these two populations undergo markedly different treatment paradigms and may require distinct pathway‐based approaches when designing new therapies. The results from our analysis show numerous pathways uniquely altered between high‐grade NMIBC and MIBC that may serve as the basis for development of alternative therapies.

## Materials and Methods

### Patient specimens

Patient tissue samples were obtained with approval by participating institution institutional review boards. High‐grade urothelial carcinoma without variant morphology was obtained from 72 patients, with matched histologically normal urothelium also obtained from 24 patients. Seven additional benign samples from patients without urothelial neoplasia history were collected. Samples were snap‐frozen, de‐identified, and stored at −80°C, and a frozen section was obtained to verify diagnosis and presence of >80% tumor nuclei. Demographic and prior treatment history was collected from patient record review. Detailed patient characteristics are described in Table**** 1. Samples were shipped via cryovial to Metabolon Inc (Durham, NC) for analysis. The analytical group was blinded to sample subgroup, and samples were, therefore, randomized on the platform for analysis.

**Table 1:** Patient demographics and precedent therapy

Cohort 1: Benign versus neoplastic comparison						
Metabolic Pathway	Normal	Nonmuscle invasive		Muscle invasive		
		Ta	T1	T2	T3	T4
Number of patients	7	5	3	2	10	4
Mean age	69.7	69.7	67.2	59.3	66.2	66.7
Gender^a^						
Male	5	5	3	2	8	4
Female	2	0	0	0	2	0
Race						
Caucasian	5	3	2	2	10	4
African‐American	2	0	0	0	0	0
Other	0	2	1	0	0	0
Prior BCG treatment		1	0	0	2	0
Prior neoadjuvant chemo^b^		0	1	0	2	2

Cohort 2: High‐grade NMIBC versus MIBC comparison					
Metabolic pathway	Nonmuscle invasive		Muscle invasive		
	High grade Ta	T1	T2	T3	T4
Number of patients	7	10	7	19	5
Mean age	67.6	69.7	74.3	64.3	68.4
Gender^a^					
Male	6	8	7	15	2
Female	1	2	0	4	3
Race					
Caucasian	7	8	5	18	5
African‐American	0	2	1	1	0
Other	0	0	1	0	0
Prior BCG treatment	1	3	0	4	0
Prior neoadjuvant chemo^b^	0	0	0	0	0

a Fishers exact test *P *=* *0.3143.

b Fishers exact test *P *=* *0.0030.

### Cell culture

T24, J82 and UM‐UC‐3 cell lines were purchased from the American Type Culture Collection (ATCC, Manassas, VA). UROtsa cells were obtained from Deutsche Sammlung fur Mikroorganismen und Zellkultur (Braunschweig, Germany). Cells were grown in RPMI‐1640 (GIBCO, Life Technologies, Grand Island, NY) supplemented with 10% fetal bovine serum (GIBCO).

### Metabolomic profiling

The mass spectrometer platforms, sample extraction and preparation, instrument settings and conditions, and data handling have been described in detail 5. In brief, weight‐normalized tissue extract containing a cocktail of recovery standards was divided into three fractions for untargeted metabolic profiling and randomized for analysis that included the following: ultrahigh‐performance liquid chromatography/tandem mass spectrometry (UHPLC‐MS/MS) in the negative ion mode, UHPLC‐MS/MS in the positive ion mode, and gas chromatography‐mass spectrometry (GC‐MS) after sialylation. Reproducibility was assessed by the recovery of the xenobiotic compounds spiked in every sample prior to extraction. The samples were analyzed using a platform consisting of a Waters ACQUITY UHPLC (Waters Corporation, Milford, MA, USA) and a Thermo‐Finnigan LTQ mass spectrometer (Thermo Fisher Scientific Inc., Waltham, MA, USA). The data extraction of the raw mass spec data files yielded information that could loaded into a relational database and manipulated without resorting to BLOB manipulation. Once in the database, the information was examined and appropriate quality control limits were imposed. Peaks were identified using Metabolon's proprietary peak integration software, and component parts were stored in a separate and specifically designed complex data structure. Compounds were identified by comparison to library entries of purified standards or recurrent unknown entities. Identification of known chemical entities was based on comparison to metabolomic library entries of purified standards. As of this writing, more than 2000 commercially available purified standard compounds had been acquired and registered into the LIMS for distribution to both the LC and GC platforms for determination of their analytical characteristics. The combination of chromatographic properties and mass spectra gave an indication of a match to the specific compound or an isobaric entity. Additional entities (unnamed compounds) were identified by their recurrent chromatographic and mass spectral signature and have the potential to be identified by future acquisition of a matching purified standard or by classical structural analysis. A variety of curation procedures were carried out to ensure that a high‐quality data set was made available for statistical analysis and data interpretation. The quality control and curation processes were designed to ensure accurate and consistent identification of true chemical entities and to remove those representing system artifacts, misassignments, and background noise. Proprietary visualization and interpretation software were used by Metabolon to confirm the consistency of peak identification among the various samples. Library matches for each compound were checked for each sample and corrected if necessary. For studies spanning multiple days, a data normalization step was performed to correct variation resulting from instrument inter‐day tuning differences. Essentially, each compound was corrected in run‐day blocks by registering the medians to equal one (1.00) and normalizing each data point proportionately (termed the “block correction”). For studies that did not require more than 1 day of analysis, no normalization is necessary, other than for purposes of data visualization.

### Statistical analysis

**Figure 1:**
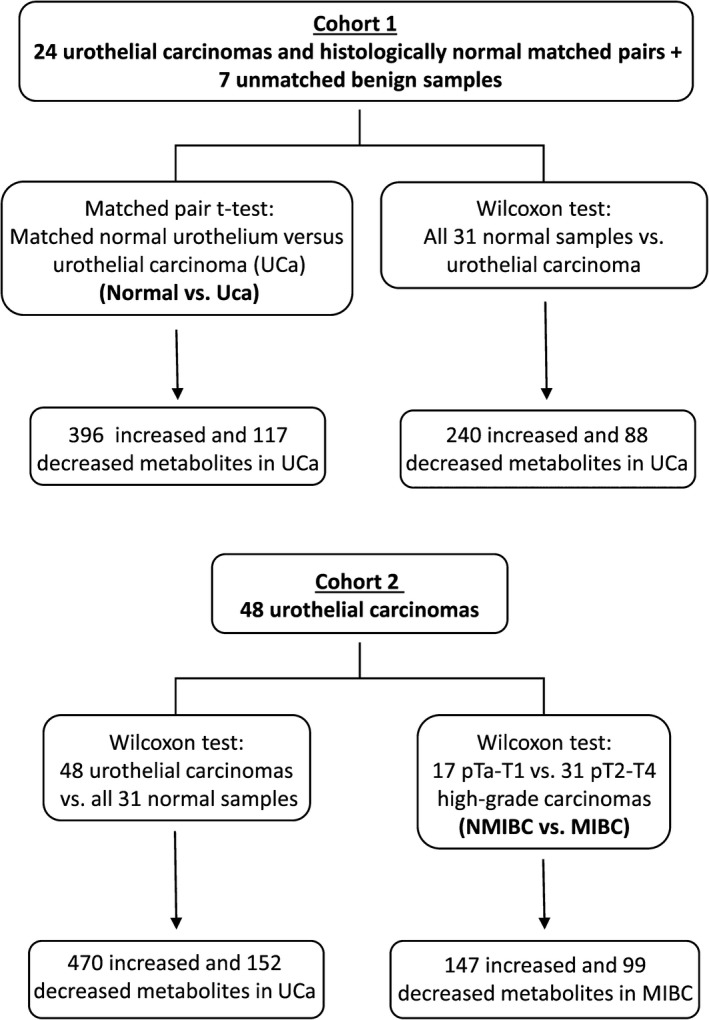
Schematic of statistical analysis using two patient cohorts of urothelial carcinoma.

All statistical analyses were performed in R version 2.14.2 (http://cran.r-project.org/). Wilcoxon test and matched‐pair t‐tests were used to determine statistical significance of metabolite mean differences between comparator groups (see Fig.**** 1 for statistical comparisons). For all analyses, missing values (if any) were imputed with the observed minimum for that particular compound (imputed values were added after block normalization). The statistical analyses were performed on natural log‐transformed data to reduce the effect of any potential outliers in the data. To account for false positives in multiple comparison testing, q‐values were utilized to estimate the false discovery rate (FDR) for a specific comparator group.

### Immunoblot analysis

Whole cell lysates were extracted with RIPA buffer containing protease and phosphatase inhibitor cocktails (Roche, Basel, Switzerland) and subjected to Western blot analysis as previously described 10. Proteins were separated on 4–15% gradient polyacrylamide–sodium dodecyl sulfate gels (Bio‐rad Laboratories, Inc., Hercules, CA), transferred to Supported Nitrocellulose membranes (Bio‐rad) using a Bio‐Rad Mini‐PROTEAN Tetracell system, followed by incubation for 1 h in a bovine serum albumin‐Tween‐20‐based blocking solution. Primary antibodies were Hexokinase I (1:1000), Hexokinase II (1:1000), phosphofructokinase (PFK) (1:1000), pyruvate kinase M1/2 isoform (PKM1/2) (1:1000), pyruvate kinase M2 isoform (PKM2) (1:1000), pyruvate dehydrogenase (PDH) (1:1000), lactate dehydrogenase A (LDHA) (1:1000), GAPDH (1:1000), and actin (1:1000), all from Cell Signaling Technology, Inc.(Danvers, MA). Blots were incubated with primary antibody overnight and then incubated for 1 h with horseradish phosphatase–conjugated anti‐rabbit or anti‐mouse secondary antibodies (1:10,000; Jackson ImmunoResearch Laboratories, Inc., West Grove, PA). Blots were developed using the Enhanced Chemiluminescence Kit (Pierce, Thermo Fisher Scientific, Waltham, MA) followed by autoradiography.

## Results

### Uniquely altered metabolites between histologically normal urothelium and high‐grade bladder cancer

To identify differences between normal‐appearing urothelium and high‐grade urothelial cancer, we used 24 matched specimens and an additional seven cases of true benign urothelium. In total, 513 metabolites were altered between normal and neoplastic tissue (matched pair t‐test, *P *≤* *0.05, FDR 3.3%), and these results held true in larger cohort of 48 urothelial carcinoma specimens, with the exception of 5 dipeptides. A detailed summary of cohort comparisons is shown in Figure 1.

#### Glucose metabolism shows a shift to glycolysis and increased shunting toward sorbitol pathway in urothelial carcinoma

**Figure 2:**
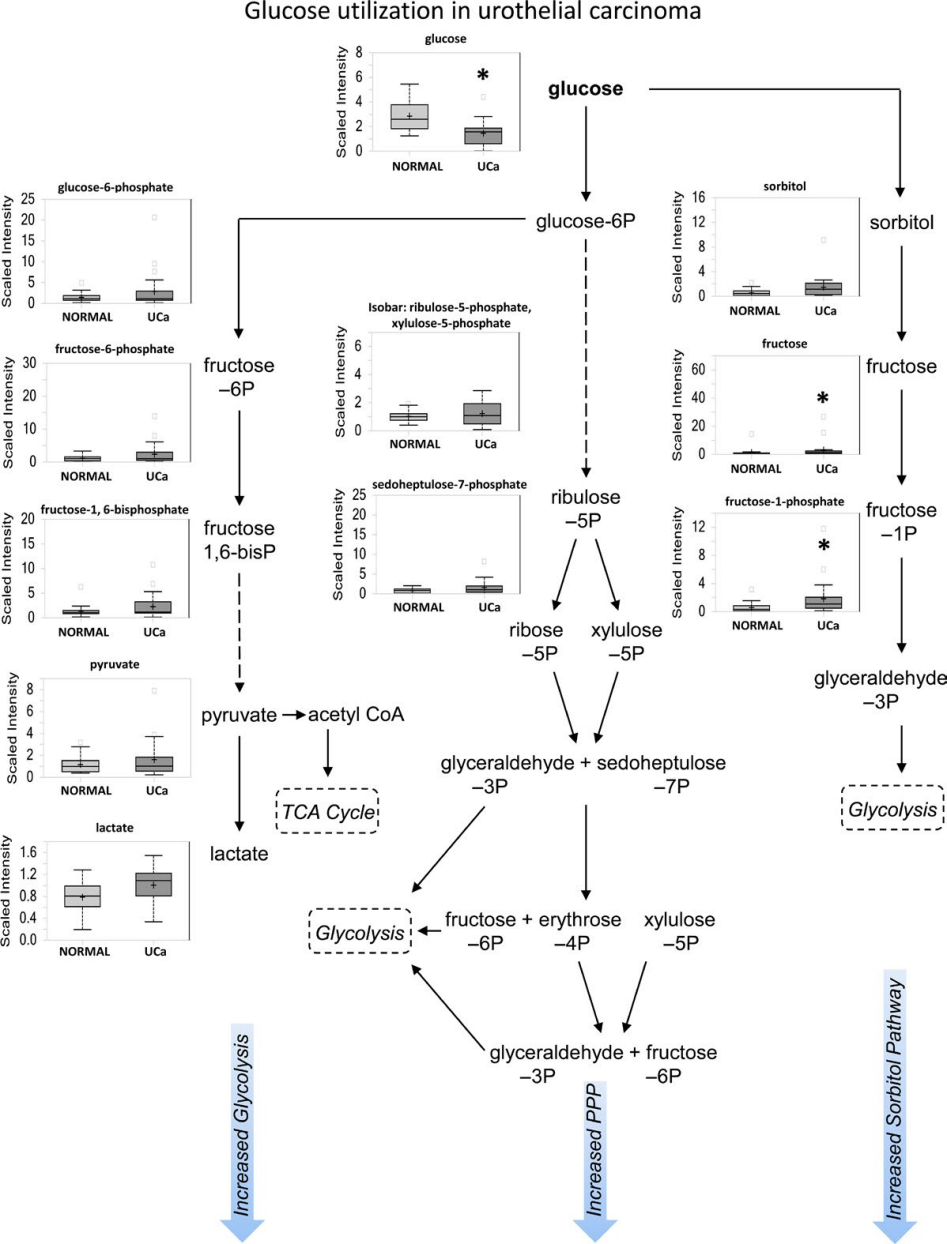
Glucose metabolism shows an increase in glycolysis, sorbitol, and PPP pathway activity in urothelial carcinoma. Box plots show a comparison between histologically normal urothelium (NORMAL) and urothelial carcinoma (UCa) matched pairs. This schematic shows glucose utilization pathways that include glycolysis, PPP, and sorbitol pathways.

A preferential shift to aerobic glycolysis (Warburg effect) occurs in many cancers, resulting in reduced glucose levels and increased pyruvate and lactate levels, among other changes 11. In contrast with normal urothelium, urothelial carcinoma showed a Warburg effect with significantly low glucose (*P *=* *0.0004) and nonsignificant increased glucose‐6‐phosphate (G6P), fructose‐6‐phosphate (F6P), fructose‐1,6‐bisphosphate (F1,6bisP), pyruvate, and lactate levels (Fig.**** 2). We validated a subset of the enzymes involved in this pathway by immunoblot analysis using a panel of bladder cells including the normal immortalized UROtsa cell line and the invasive T24, J82, and UM‐UC‐3 lines. Hexokinase II, PFK, PDH, and LDHA protein expression are increased in invasive bladder cancer cells compared with UROtsa cells (Fig. S1).

Rather than continuing on the glycolytic pathway, the G6P metabolite can instead be routed to the pentose phosphate pathway (PPP) for regeneration of reduced nicotinamide adenine dinucleotide phosphate (NADPH) to maintain redox status. Although slight increases in late PPP intermediates such as ribulose‐5‐phosphate (Ru5P), sedoheptulose‐7‐phosphate (S7P), and fructose‐6‐phosphate (F6P) were observed, only the end‐product ribose was significantly increased in neoplastic urothelium (*P *=* *0.0008), suggesting activation of the PPP pathway. Glucose may also be shunted into the sorbitol pathway, which appears to be enhanced in urothelial neoplasia as well, with nonsignificant elevation of sorbitol and significantly increased downstream derivatives fructose (*P *=* *0.0001) and fructose‐1‐phosphate (*P *=* *0.0028).

#### Increased late‐stage TCA cycle intermediates indicate anaplerotic activity in urothelial carcinoma

**Figure 3:**
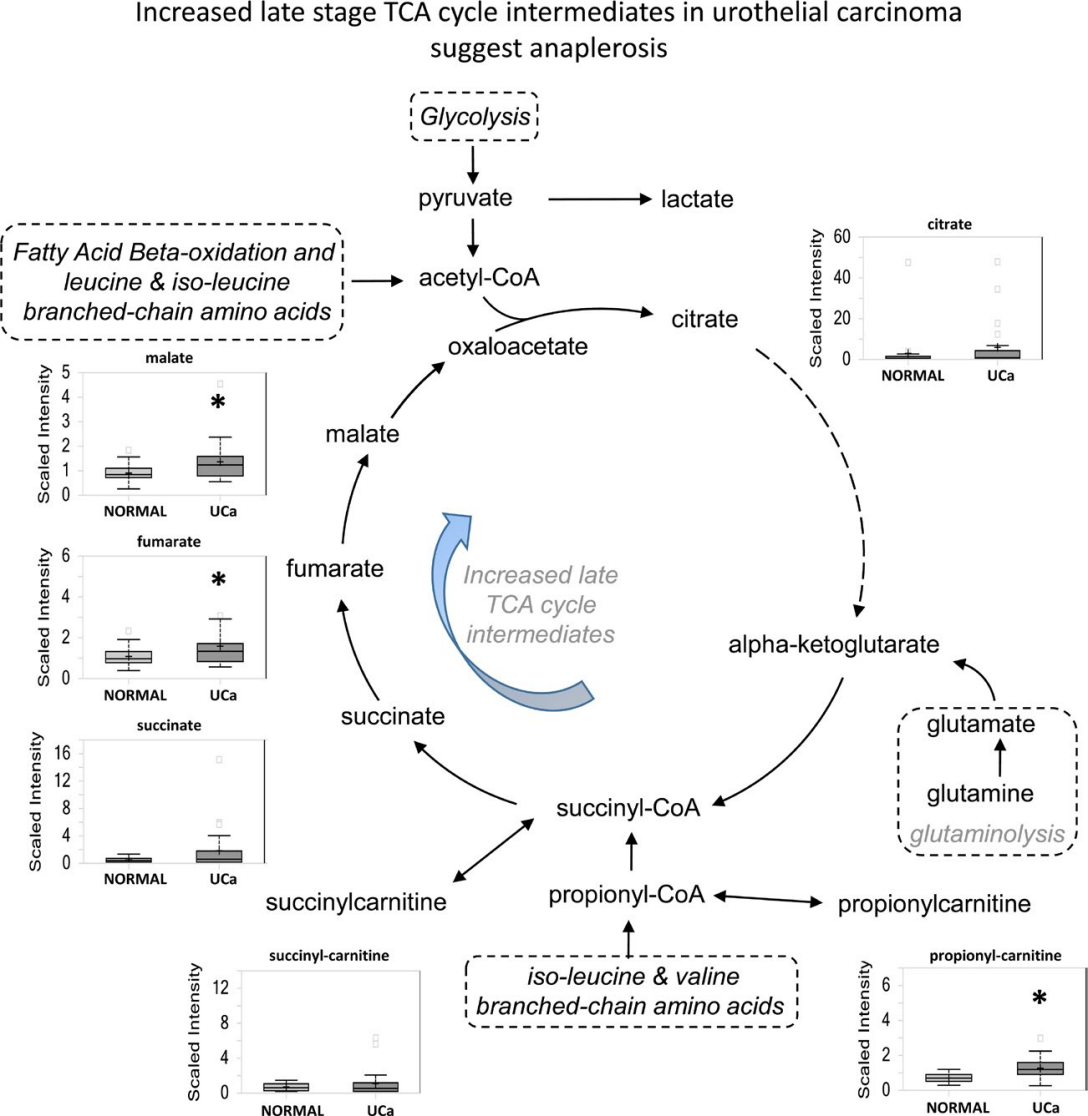
Increased late‐stage TCA cycle intermediates suggest the potential for increased anaplerotic activity to sustain energy production in urothelial carcinoma. Elevations in metabolites are seen in carcinoma specimens suggest a potential role for anaplerosis in UCa.

Pyruvate generated by glycolysis enters mitochondria for conversion to acetyl‐CoA, which then reacts with oxaloacetate to form citrate. Citrate can proceed through the TCA cycle or alternatively be transported to the cytoplasm for fatty acid synthesis. We identify significant increases in late TCA cycle intermediates fumarate (*P *=* *0.0269) and malate (*P *=* *0.0249) and nonsignificant increase in citrate and succinate in urothelial carcinoma (Fig. 3). This increase, when coupled with higher levels of amino acids described later in this study, suggests anaplerotic activity may occur in urothelial carcinoma as a mechanism to sustain energy production.

#### Lipid metabolism shows formation of fatty acids, glycerophospholipids, and sphingolipids in urothelial carcinoma

**Figure 4:**
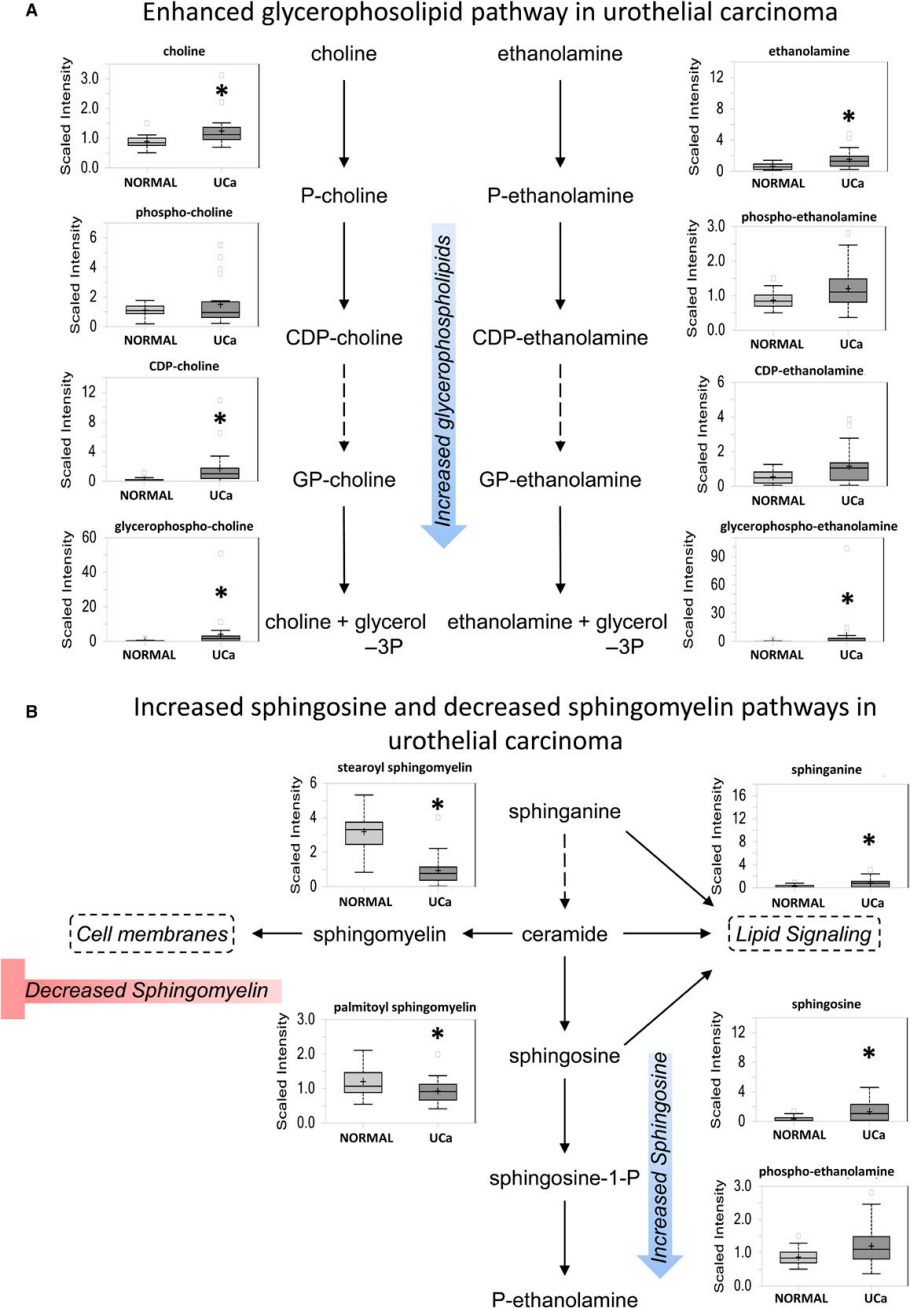
Glycerophospholipid and sphingolipid pathways are shown as representatives of lipid metabolism pathways altered in urothelial carcinoma. (A) Most glycerophospholipids are elevated in UCa. (B) Sphingolipid metabolism shows increased sphingosine synthesis at the cost of sphingomyelin.

Urothelial carcinoma shows significant upregulation of multiple lipid classes in contrast with normal urothelium that include medium chain, long chain, polyunsaturated and branched fatty acids, monoacylglycerols, lysolipids, and endocannabinoids (*P *<* *0.05 for all classes). Significant elevations of glycerophospholipids (Fig. 4A) and sphingolipids (Fig. 4B), which are critical components of cell membrane biosynthesis and turnover during cell proliferation, were present in urothelial carcinoma**.** The significant increase in sphinganine (*P *=* *0.0149) and sphingosine (*P *=* *0.0364), with concurrent significant decrease in sphingomyelin derivatives stearoyl sphingomyelin (*P *=* *8.52E‐07) and palmitoyl sphingomyelin (*P *=* *0.0069) suggests preferential conversion of ceramide to sphingosine in cancer cells (Fig. 4B).

Additional upregulated long‐chain acyl‐carnitines include propionylcarnitine, valerylcarnitine, and deoxycarnitine, which, when combined with a slight increase of ketone body beta‐hydroxybutyrate (BHBA) and decreased dicarboxylic fatty acids, suggest efficient beta‐oxidation of fatty acids in urothelial carcinoma.

#### Elevated amino acid metabolism also supports a potential anaplerotic mechanism in urothelial carcinoma

**Figure 5:**
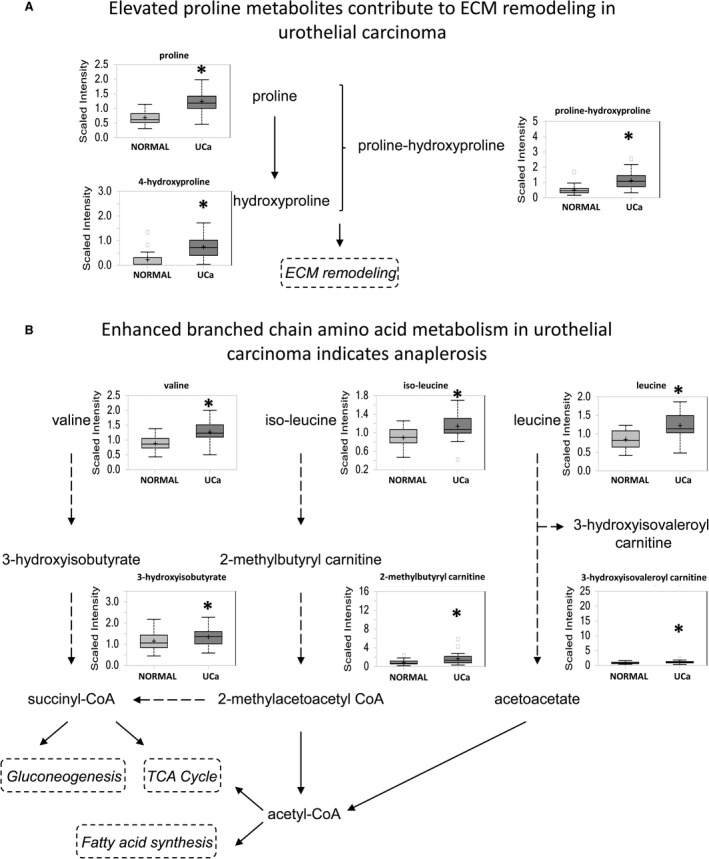
Elevated amino acid metabolism supports the potential role of anaplerosis in urothelial carcinoma energy production. (A) One of the highest increases were seen with proline which is a component of the ECM. (B) Increased production of branched chain amino acids valine, iso‐leucine, and leucine was seen.

Most amino acids were significantly elevated in urothelial carcinoma, with highest increases in proline (*P *=* *5.92E‐09), alanine (*P *=* *1.09E‐05), and asparagine (*P *=* *2.60E‐06). Increased amino acid levels reflect either increased protein breakdown within cancer cells or increased uptake of amino acids into cancer cells from the microenvironment. Proline and its derivatives are important constituents of collagen, which makes up the extracellular matrix, and were significantly upregulated in urothelial carcinoma (Fig. 5A). Other extracellular matrix‐related metabolites were also increased, including glucosamine (*P *=* *0.0118), N‐acetylglucosamine (*P *=* *0.0003), N‐acetylglucosamine 6‐phosphate (*P *=* *0.0007), N‐acetylgalactosamine (*P *=* *6.95E‐06), N‐acetylneuraminate (*P *=* *2.25E‐05), fucose (*P *=* *0.0075), erythronate (*P *=* *5.84E‐05), glucuronate (*P *=* *0.0142), and UDP‐glucuronate (*P *=* *0.0157), which may indicate active extracellular matrix remodeling in the context of carcinoma development and/or progression. Furthermore, nutrient stress can promote alpha‐ketoglutarate and glutamate formation by proline shunting to proline oxidase, which contributes to anaplerosis 12, 13.

Branched‐chain amino acid metabolism was also increased in urothelial carcinoma, including increased leucine (*P *=* *3.80E‐06), isoleucine (*P *=* *0.0002), valine (*P *=* *2.07E‐07), and their degradation products (Fig. 5B). Enhanced catabolism and anaplerosis may contribute to the observed changes in the late TCA intermediates, supported by higher levels of propionylcarnitine.

#### Purine/pyrimidine metabolism shows enhanced production of deoxy‐nucleotides and derivation of purine from de novo synthesis and catabolism in cancer

Both purine and pyrimidine metabolites were increased in urothelial carcinoma, which, when combined with increased catabolic products, suggests increased degradation of nucleic acids (Table**** 2). Methylated nucleobases were also globally increased and may be caused by either enhanced nucleic acid turnover or increased methylation 14. Purine levels are increased due to both, de novo synthesis and catabolism of nucleotides. The decreased levels of adenosine, inosine, and guanosine, coupled with elevated levels of guanine, hypoxanthine, and xanthine, could be the result of potentially elevated xanthine oxidase levels leading to increased oxidative stress 15. Most bases, nucleosides, and nucleotides of the pyrimidine metabolic pathway were also increased in urothelial carcinoma.

**Table 2:** Alterations in purine metabolites

Class	Metabolite	Carcinoma:normal ratio	*P*‐value
Bases	Xanthine	2.01	0.0003
	Hypoxanthine	1.42	0.0008
	Adenine	1.04	0.1697
	Guanine	1.76	0.8688
Nucleosides	Xanthosine	1.09	0.3383
	Inosine	0.7	0.0018
	2′‐deoxyinosine	3.42	7.72E‐05
	Adenosine	0.6	0.0434
	Guanosine	0.77	0.0122
	2′‐deoxyguanosine	1.9	0.0007
Nucleotides	Inosine 5′‐monophosphate	0.84	0.3246
	Adenosine 2′‐monophosphate	2.72	0.0008
	Adenosine 3′‐monophosphate	4.64	6.50E‐06
	Adenosine 5′‐monophosphate	1.3	0.7892
	Adenosine 5′‐diphosphate	0.65	0.07
	Guanosine 5′‐monophosphate	2.42	0.4561
	Guanosine 3′‐monophosphate	5.8	4.05E‐05
Methylated	N1‐methyladenosine	1.68	0.0005
	7‐methyguanine	1.88	7.51E‐05
	N1‐methylguanosine	3.7	2.28E‐07
	N2‐methyladenosine	2.03	4.72E‐05
	N2, N2‐dimethylguanosine	2.67	1.04E‐06
Biosynthesis	AICA ribonucleotide	2	0.0078
	Adenylosuccinate	6.22	0.0044
Catabolism	Urate	1.59	0.7592
	Allantoin	3.32	0.9491

### Differential metabolites between nonmuscle‐invasive and muscle‐invasive urothelial carcinoma suggest possible uniquely actionable pathways

We compared data between NMIBC and MIBC using 48 pathologically staged samples (Fig. 1). Overall 246 metabolites were distinctly present between NMIBC and MIBC samples (Wilcoxon test, *P *≤* *0.05, FDR 11.3%).

#### Elevated prostaglandin and thromboxane levels occur in pathologically advanced disease

**Figure 6:**
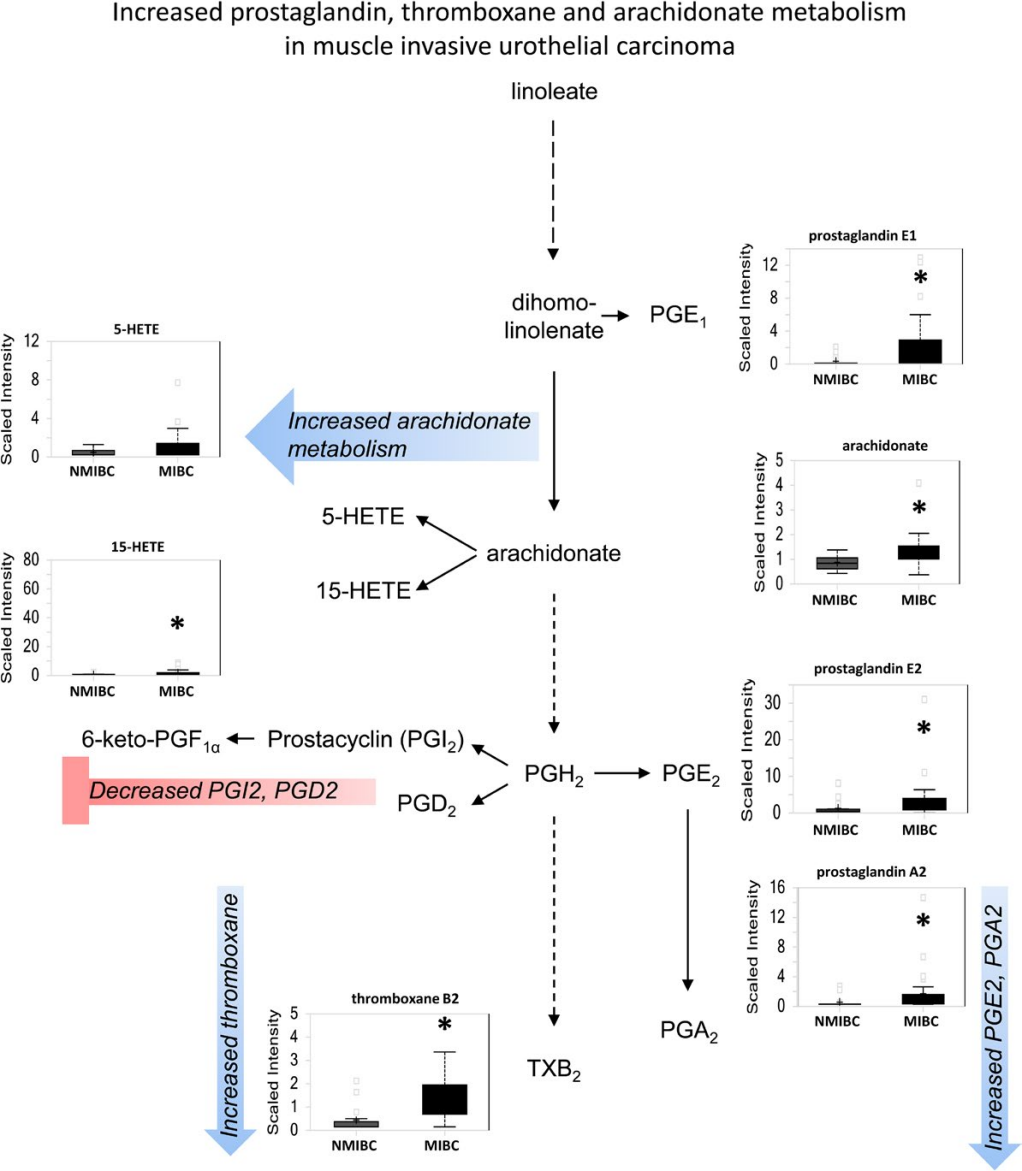
Eicosanoid metabolism is preferentially increased in MIBC as compared to NMIBC. The de novo pathway of NAD+ synthesis is enhanced, whereas the salvage pathway is repressed in more advanced urothelial carcinoma. Increased activity of COX and LOX pathways suggests a potential role for inflammatory mediators in MIBC.

Eicosanoids such as prostaglandins, thromboxanes, and leukotrienes are biologically active signaling components of the COX and LOX pathways. Elevated prostaglandin A2 (*P *=* *0.002), B2 (*P *=* *0.025), E1 (*P *=* *0.004), E2 (*P *=* *0.0066) (PGA2, PGB2, PGE1, PGE2, respectively), and thromboxane B2 (*P *=* *0.002) occur in MIBC in contrast to both normal urothelium and NMIBC (Fig. 6), whereas PGD2, PGI2, and 6‐keto prostaglandin F1alpha were significantly decreased in urothelial carcinoma in contrast to normal urothelium (data not shown). These findings suggest that urothelial carcinomas that are at least T2 in nature show enhanced COX, prostaglandin synthase E, and thromboxane synthase pathways and repressed prostaglandin synthase D and prostacyclin synthase pathways. In addition, increased arachidonate metabolism occurs in MIBC, with evidence of elevated 5‐HETE (*P *=* *0.0747) and 15‐HETE (*P *=* *0.0147).

#### MIBC derives NAD+ from tryptophan instead of the salvage pathway

**Figure 7:**
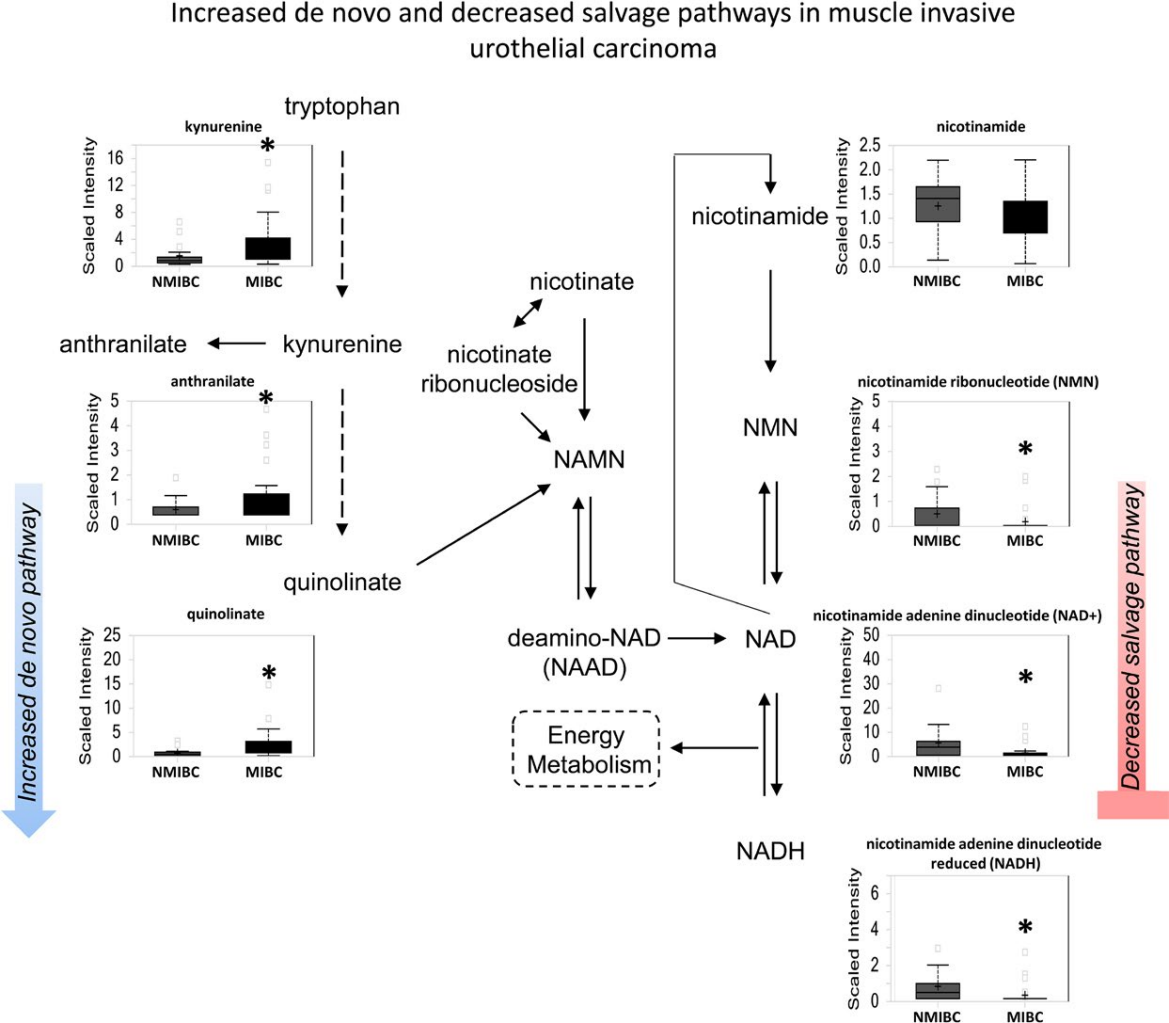
NAD+ metabolism is elevated in MIBC as compared with NMIBC. The de novo pathway of NAD+ synthesis is enhanced, whereas the salvage pathway is repressed in more advanced urothelial carcinoma. Increased of kynurenine, 3‐hydroxykyurenine, and quinolinate suggest a potential role for inflammatory mediators in MIBC.

NAD+ is produced either by de novo synthesis from tryptophan or the salvage pathway from nicotinamide and nicotinate. Elevated kynurenine (*P *=* *0.0212), anthranilate (*P *=* *0.0111), and quinolate (*P *=* *0.0015) in MIBC indicate that de novo synthesis of NAD+ occurs in MIBC (Fig.**** 7). This may be influenced by local TNF‐alpha and INF‐gamma production by local inflammatory cells and requires further investigation 16. Furthermore, the salvage pathway for NAD+ synthesis appears defective in MIBC, evidenced by reduced levels of NMN (*P *=* *0.0239), NAD+ (*P *=* *0.0062), and NADH (*P *=* *0.0007).

#### Hemoglobin catabolites are increased in MIBC

The hemoglobin catabolite bilirubin was significantly higher in MIBC (*P *=* *0.0001 for bilirubin (Z,Z) and *P *=* *0.0069 for bilirubin (E,E)), suggesting enhanced heme catabolism in higher stage tumors.

## Discussion

Metabolomics is an emerging “omics” technology which gives information about the sum total of all metabolites within a biological sample which represents the end result of biological processes as a manifestation of its genetic information. It is a functional readout of the tissue biochemistry that better mirrors the phenotypic state as compared to other approaches such as transcriptomics and proteomics. The application of metabolomic analysis in bladder cancer has primarily focused on the distinction between normal‐appearing urothelium and bladder cancer tissue and on the identification of bladder cancer using serum and urine samples [5, 6]. We have expanded upon studies in the literature by including the analysis of urothelial carcinomas in different stage categories, using the distinction between NMIBC and MIBC as a primary stratifier given the unique treatment modalities for these two populations. We confirmed unique pathway alterations that differentiate normal‐appearing urothelium from urothelial carcinoma in two different cohorts and secondarily stratified metabolomic data between NMIBC and MIBC. Many major metabolic pathways were altered between normal‐appearing urothelium and urothelial carcinoma, whereas a more limited metabolite profile distinguished NMIBC from MIBC.

Metabolites associated with cellular energy status were frequently altered in urothelial carcinoma. Preferential use of glucose for glycolysis and sorbitol production was identified in urothelial carcinoma and is indicative of the Warburg phenomenon that is common in cancer 11. Anaplerosis, a mechanism to replenish TCA cycle intermediates such as oxaloacetate, alpha‐ketoglutarate, succinyl‐CoA, and fumarate from substrates such as amino acids and fatty acids 17, also appears to occur in urothelial carcinoma to maintain energy status in cancer cells. Evidence in support of anaplerotic activity includes elevation of late‐stage TCA intermediates, increased amino acid metabolites such as succinylcarnitine and propionylcarnitine, and increased fatty acid beta‐oxidation to generate acetyl‐CoA and succinyl‐CoA.

Increased production of the majority of lipid classes occurs in urothelial carcinoma and can influence numerous cancer‐related processes that include membrane formation, energy metabolism precursors, and cellular signaling 18. Increased lipid classes may occur through several mechanisms. One mechanism is increased biosynthesis from citrate, which has been reported to be increased in urothelial carcinoma 11. A second mechanism to increase lipid levels is an increased rate of lipid membrane turnover and membrane remodeling that may occur secondary to increased cancer cell proliferation. Finally, inflammatory infiltrates may also induce cancer cell lipid membrane turnover, resulting in higher levels of fatty acids 18. In our study, elevation in lysolipid levels in urothelial carcinoma may support a role for membrane turnover in lipid elevation, rather than derivation from citrate.

We also identified changes in sphingolipid metabolism in urothelial carcinoma. Specifically, increased sphingosine and reduced sphingomyelin formation in cancer is consistent with activity of sphingosine‐1‐phosphate that has been implicated in cancer growth 19. Decreases in sphingomyelin intermediates seen in urothelial carcinoma may occur secondary to increased sphingomyelin breakdown, decreased sphingomyelin synthesis, or increased microvesicle formation in carcinoma, with the latter reported to critically alter tumor biology by regulating cell‐to‐cell communication and influence invasion, inflammation, expansion, etc 20. As significant advances in sphingolipid‐based cancer therapeutics have been reported 21, further analysis of this pathway in urothelial carcinoma therapeutics may be warranted.

Finally, we identified several changes of relevance to nucleotide synthesis in urothelial carcinoma relative to normal‐appearing urothelium. Shunting of glucose to the PPP produces ribose 5‐phosphate, a major 5‐sugar moiety required for nucleotide synthesis 22. We also identified increased purine and pyrimidine pathway intermediates in urothelial carcinoma. When combined with data from energy pathways, this suggests that proliferative capacity in urothelial carcinoma is likely fueled through both glucose and TCA cycle intermediate catabolism.

We next compared metabolite differences between high‐grade NMIBC and MIBC, an analysis that is unique to our metabolomic study on urothelial carcinoma. Although these broad classification categories encompass carcinomas at different stages and thus in the progression spectrum of urothelial carcinoma, we selected this stratification based on the distinct treatment approaches between these two categories and the need for alternative therapies specific to these two settings. Three major pathways were uniquely altered in MIBC and included increased eicosanoid signaling, enhanced use of de novo synthesis of NAD+ for energy and DNA repair, and increased heme catabolism.

Altered eicosanoid metabolites seen in MIBC suggest an increase in both COX and LOX activity, with alterations in numerous metabolites in these pathways. These pathways have been implicated in inflammatory cell regulation, tumorigenesis, cell proliferation, and angiogenesis and represent potential actionable pathways in urothelial carcinoma 23, 24, 25. The COX pathway can have mixed effects on tumorigenesis, with COX and PGE2 implicated in pro‐tumorigenic activity, whereas PGD2 and PGI2 may have anti‐tumorigenic activities 26, 27. Increased LOX‐mediated arachidonate metabolism was also evident in our study and is supported by a recent description of increased 5‐LOX in urothelial carcinoma relative to normal urothelium 23. 5‐LOX activity is essential for urothelial carcinoma cell growth and requires the 5‐LOX product 5‐HETE 24, which we also identified as increased in MIBC.

We also found increased NAD+ de novo synthesis in MIBC and prior reports have suggested that cancers highly dependent on NAD^+^ can use this metabolite as a cofactor for the DNA repair enzyme PARP 28. Finally, elevated levels of hemoglobin catabolites were identified in MIBC, with putative increased heme oxygenase‐1 (HO‐1) activity responsible for increased heme cleavage to biliverdin and ultimately bilirubin. HO‐1 upregulation occurs in cancer and regulates oncogenic factors, such as matrix metalloproteinase (MMP) family members and vascular endothelial growth factor (VEGF) 25. HO‐1 is also upregulated by smoking, a major risk factor for urothelial carcinoma 29. Although no HO‐1 targeting drugs are currently available, many novel HO‐1 inhibitors are being developed and may be of relevance to urothelial carcinoma 30.

Two major effectors that could influence regulation of these multiple pathways in parallel include both p53 activity and associated inflammation. Although analysis of these two mechanisms was beyond the scope of the current analysis, both factors could potentially play a significant role in coordinating the metabolic changes we have identified. The tumor‐suppressor p53 is frequently mutated in bladder cancer 3, 11 and can regulate glucose metabolism, lipid metabolism, oxidative stress, proline oxidase expression, and apoptosis through effects on peroxisome proliferator‐activated receptor gamma (PPAR‐*γ*) 12, 13, 31, 32. Inflammation has also been implicated in cancer cell behavior, and we have identified several alterations that suggest inflammation status may impact changes in urothelial carcinoma. Specifically, pathway alterations present in fatty acid production, proline synthesis, NAD+ synthesis, and heme catabolism can all be directionally regulated by inflammatory cells 33, 34, 35, 36. Given that the vast majority of patients in this study are BCG‐naïve, many of these effects may relate to underlying tumor cell and/or stromal factors and appear to be especially pronounced in MIBC 37.

We compared our results to two prior studies, which have primarily focused on the distinction between benign and cancerous tissue, rather than stage or progression. Putluri et al. 8 identified 35 biochemicals differentiating benign and tumor. We identified all but eight of these. Of those that were present, 19 showed similar directional change, one was unaltered and seven showed opposite directional change. Tripathi et al. 4 identified eight differential metabolites between normal and tumor using nuclear magnetic resonance. All of them showed the same directional change in our study. In contrast to their study, which did not find differences between pTa/pT1 and >pT2 tumors, our analysis revealed several significantly altered metabolites. Furthermore, in contrast to both studies, our analysis, through the utilization of an unbiased global biochemical profiling LC/MS and GC/MS based platform employing a biochemical library of greater than 2000 known biochemicals, resulted in the detection of hundreds of significantly altered metabolites that are consistent with the cancer phenotype reported both in bladder cancer and other solid tumors. One limitation in all studies that employ normal‐appearing (“benign”) urothelium in analysis of bladder cancer patients is that preneoplastic molecular alterations may be present without affecting the morphological appearance of the urothelium. Given that all studies evaluating normal‐appearing urothelium and bladder carcinoma to date have identified similar alterations in numerous metabolite categories, this limitation may be less critical. Our study has also expanded on prior analyses to include more than one patient cohort and to analyze differences in metabolites between NMIBC and MIBC. We also compared our results to a recent bladder cancer urine study which reported six biomarkers that differentiated cancer and noncancer samples 5. Three of them had the same directional change in our study, which suggests that changes in these metabolites are due to cancer. Based on these analyses, several potential novel therapeutics approaches can be proposed pending further analysis (Table 3).

**Table 3:** Selected list of potential actionable targets in urothelial carcinoma identified by metabolomic analysis

Pathway	Target molecule	Agent
PPAR	PPAR*γ* Dual PPAR*γ* and PPAR*α*	Thiazolidinediones (Rosiglitazone, Pioglitazone) Muraglitazar, Tesaglitazar
Sphingolipid	Glucosyl ceramide synthase Sphingosine kinase Sphingosine 1‐phosphate receptor Ceramidase	PPMP, PPPP, etc. SKi, DHS, etc. FTY‐720, LT1002, etc. D‐MAPP, B13, etc.
Eicosanoid	LOX‐5 COX‐2 Dual COX‐2 and COX‐1 Dual LOX‐5 and COX‐2	AA861 Celecoxib, Rofecoxib NSAIDs (ibuprofen, aspirin, etc.) Licofelone
Heme	HO‐1	Metalloporphyrins (ZnPP, SnMP etc.) Imidazole derivatives (Azalanstat, OB‐24)

Limitations of our study include small numbers of Cohort 1 samples which did not allow for NMIBC versus MIBC comparisons and lack of matched urine samples. Despite these limitations, however, the data maintain robustness across two cohorts with minor metabolite shifts. Follow‐up studies that incorporate larger patient cohorts using matched samples and functional testing would be necessary to confirm our results. Correlation of metabolomics data with smoking status, p53 expression, treatments, and outcomes would be valuable. Use of other complementary –omics analyses with metabolomics would provide greater information. Review of TCGA data suggests that many of our altered pathways may also show dysregulation at the transcriptomic level. Studies using a single patient dataset that evaluates both signatures concordantly, combined with functional studies, may be better to assess whether transcriptional changes are responsible for metabolic pathway alterations. We also did not have adequate amounts of carcinoma in situ (Cis) frozen samples and future studies which include adequate volumes of these samples can further elaborate on changes in this group.

In conclusion, we used a highly sensitive metabolomics approach to identify multiple metabolomic pathway changes in urothelial carcinoma. Both glycolysis and anaplerosis appear to be important mechanisms to maintain energy status in urothelial carcinoma. MIBC appears to be distinct in its enhanced signaling through COX and LOX pathways, NAD+ synthesis regulation, and heme catabolism. Many of the differential pathways suggest unique actionable approaches to NMIBC and MIBC, pending further investigation.

## Conflict of Interest

BW is an employee and BN is a retired employee of Metabolon Inc.

## Supporting information


**Figure S1.** Immunoblot validation of glucose metabolism enzymes shows altered protein levels that correspond to metabolomics data. Benign UROtsa and invasive T24, J82, and UM‐UC‐3 cell lines are shown.

## References

[1] Ferlay, J. , I. Soerjomataram , R. Dikshit , S. Eser , C. Mathers , M. Rebelo , et al. 2015 Cancer incidence and mortality worldwide: sources, methods and major patterns in GLOBOCAN 2012. Int. J. Cancer 136:E359–E386. 25220842 10.1002/ijc.29210

[2] Siegel, R. L. , K. D. Miller , and A. Jemal . 2015 Cancer statistics, 2015. CA Cancer J. Clin. 65:5–29. 25559415 10.3322/caac.21254

[3] Kamat, A. M. , and P. Mathew . 2011 Bladder cancer: imperatives for personalized medicine. Oncology (Williston Park) 25:951–958, 960. 22010395

[4] Tripathi, P. , B. S. Somashekar , M. Ponnusamy , A. Gursky , S. Dailey , P. Kunju , et al. 2013 HR‐MAS NMR tissue metabolomic signatures cross‐validated by mass spectrometry distinguish bladder cancer from benign disease. J. Proteome Res. 12:3519–3528. 23731241 10.1021/pr4004135 PMC3722911

[5] Wittmann, B. M. , S. M. Stirdivant , M. W. Mitchell , J. E. Wulff , J. E. McDunn , Z. Li , et al. 2014 Bladder cancer biomarker discovery using global metabolomic profiling of urine. PLoS ONE 9:e115870. 25541698 10.1371/journal.pone.0115870 PMC4277370

[6] Bansal, N. , A. Gupta , N. Mitash , P. S. Shakya , A. Mandhani , A. A. Mahdi , et al. 2013 Low‐ and high‐grade bladder cancer determination via human serum‐based metabolomics approach. J. Proteome Res. 12:5839–5850. 24219689 10.1021/pr400859w

[7] Dettmer, K. , F. C. Vogl , A. P. Ritter , et al. 2013 Distinct metabolic differences between various human cancer and primary cells. Electrophoresis 34:2836–2847. 23857076 10.1002/elps.201300228

[8] Putluri, N. , A. Shojaie , V. T. Vasu , S. K. Vareed , S. Nalluri , V. Putluri , et al. 2011 Metabolomic profiling reveals potential markers and bioprocesses altered in bladder cancer progression. Cancer Res. 71:7376–7386. 21990318 10.1158/0008-5472.CAN-11-1154 PMC3249241

[9] von Rundstedt, F. C. , K. Rajapakshe , J. Ma , J. M. Arnold , J. Gohlke , V. Putluri , et al. 2016 Integrative Pathway Analysis of Metabolic Signature in Bladder Cancer: a Linkage to The Cancer Genome Atlas Project and Prediction of Survival. J. Urol. 195:1911–1919. 26802582 10.1016/j.juro.2016.01.039 PMC4861129

[10] Gupta, S. , A. M. Hau , J. R. Beach , J. Harwalker , E. Mantuano , S. L. Gonias , et al. 2013 Mammalian target of rapamycin complex 2 (mTORC2) is a critical determinant of bladder cancer invasion. PLoS ONE 8:e81081. 24312263 10.1371/journal.pone.0081081 PMC3842329

[11] Levine, A. J. , and A. M. Puzio‐Kuter . 2010 The control of the metabolic switch in cancers by oncogenes and tumor suppressor genes. Science 330:1340–1344. 21127244 10.1126/science.1193494

[12] Pandhare, J. , S. P. Donald , S. K. Cooper , and J. M. Phang . 2009 Regulation and function of proline oxidase under nutrient stress. J. Cell. Biochem. 107:759–768. 19415679 10.1002/jcb.22174 PMC2801574

[13] Liu, Y. , G. L. Borchert , S. P. Donald , B. A. Diwan , M. Anver , and J. M. Phang . 2009 Proline oxidase functions as a mitochondrial tumor suppressor in human cancers. Cancer Res. 69:6414–6422. 19654292 10.1158/0008-5472.CAN-09-1223 PMC4287397

[14] Porcelli, B. , L. F. Muraca , B. Frosi , E. Marinello , R. Vernillo , A. De Martino , et al. 1997 Fast‐atom bombardment mass spectrometry for mapping of endogenous methylated purine bases in urine extracts. Rapid Commun. Mass Spectrom. 11:398–404. 9069642 10.1002/(SICI)1097-0231(19970228)11:4<398::AID-RCM807>3.0.CO;2-M

[15] Klaunig, J. E. , L. M. Kamendulis , and B. A. Hocevar . 2010 Oxidative stress and oxidative damage in carcinogenesis. Toxicol. Pathol. 38:96–109. 20019356 10.1177/0192623309356453

[16] Chiarugi, A. , M. Calvani , E. Meli , E. Traggiai , and F. Moroni . 2001 Synthesis and release of neurotoxic kynurenine metabolites by human monocyte‐derived macrophages. J. Neuroimmunol. 120:190–198. 11694334 10.1016/s0165-5728(01)00418-0

[17] Owen, O. E. , S. C. Kalhan , and R. W. Hanson . 2002 The key role of anaplerosis and cataplerosis for citric acid cycle function. J. Biol. Chem. 277:30409–30412. 12087111 10.1074/jbc.R200006200

[18] Cerne, D. , I. P. Zitnik , and M. Sok . 2010 Increased fatty acid synthase activity in non‐small cell lung cancer tissue is a weaker predictor of shorter patient survival than increased lipoprotein lipase activity. Arch. Med. Res. 41:405–409. 21044743 10.1016/j.arcmed.2010.08.007

[19] Ponnusamy, S. , M. Meyers‐Needham , C. E. Senkal , S. A. Saddoughi , D. Sentelle , S. P. Selvam , et al. 2010 Sphingolipids and cancer: ceramide and sphingosine‐1‐phosphate in the regulation of cell death and drug resistance. Future Oncol. 6:1603–1624. 21062159 10.2217/fon.10.116 PMC3071292

[20] Muralidharan‐Chari, V. , J. W. Clancy , A. Sedgwick , and C. D'Souza‐Schorey . 2010 Microvesicles: mediators of extracellular communication during cancer progression. J. Cell Sci. 123:1603–1611. 20445011 10.1242/jcs.064386 PMC2864708

[21] Adan‐Gokbulut, A. , M. Kartal‐Yandim , G. Iskender , and Y. Baran . 2013 Novel agents targeting bioactive sphingolipids for the treatment of cancer. Curr. Med. Chem. 20:108–122. 23244584

[22] Tong, X. , F. Zhao , and C. B. Thompson . 2009 The molecular determinants of de novo nucleotide biosynthesis in cancer cells. Curr. Opin. Genet. Dev. 19:32–37. 19201187 10.1016/j.gde.2009.01.002 PMC2707261

[23] Madka, V. , A. Mohammed , Q. Li , Y. Zhang , J. M. Patlolla , L. Biddick , et al. 2014 Chemoprevention of urothelial cell carcinoma growth and invasion by the dual COX‐LOX inhibitor licofelone in UPII‐SV40T transgenic mice. Cancer Prev. Res. (Phila.) 7:708–716. 24795386 10.1158/1940-6207.CAPR-14-0087 PMC4310686

[24] Hayashi, T. , K. Nishiyama , and T. Shirahama . 2006 Inhibition of 5‐lipoxygenase pathway suppresses the growth of bladder cancer cells. Int. J. Urol. 13:1086–1091. 16903934 10.1111/j.1442-2042.2006.01485.x

[25] Miyata, Y. , S. Kanda , K. Mitsunari , A. Asai , and H. Sakai . 2014 Heme oxygenase‐1 expression is associated with tumor aggressiveness and outcomes in patients with bladder cancer: a correlation with smoking intensity. Transl. Res. 164:468–476. 25063314 10.1016/j.trsl.2014.06.010

[26] Keith, R. L. , Y. E. Miller , T. M. Hudish , C. E. Girod , S. Sotto‐Santiago , W. A. Franklin , et al. 2004 Pulmonary prostacyclin synthase overexpression chemoprevents tobacco smoke lung carcinogenesis in mice. Cancer Res. 64:5897–5904. 15313935 10.1158/0008-5472.CAN-04-1070

[27] Kim, J. , P. Yang , M. Suraokar , A. L. Sabichi , N. D. Llansa , G. Mendoza , et al. 2005 Suppression of prostate tumor cell growth by stromal cell prostaglandin D synthase‐derived products. Cancer Res. 65:6189–6198. 16024620 10.1158/0008-5472.CAN-04-4439

[28] Weaver, A. N. , and E. S. Yang . 2013 Beyond DNA Repair: additional Functions of PARP‐1 in Cancer. Front Oncol. 3:290. 24350055 10.3389/fonc.2013.00290 PMC3841914

[29] Besaratinia, A. , and S. Tommasi . 2013 Genotoxicity of tobacco smoke‐derived aromatic amines and bladder cancer: current state of knowledge and future research directions. FASEB J. 27:2090–2100. 23449930 10.1096/fj.12-227074

[30] Motterlini, R. , and R. Foresti . 2014 Heme oxygenase‐1 as a target for drug discovery. Antioxid. Redox Signal. 20:1810–1826. 24180608 10.1089/ars.2013.5658

[31] Liang, Y. , J. Liu , and Z. Feng . 2013 The regulation of cellular metabolism by tumor suppressor p53. Cell Biosci. 3:9. 23388203 10.1186/2045-3701-3-9 PMC3573943

[32] Kim, K. Y. , J. H. Ahn , and H. G. Cheon . 2007 Apoptotic action of peroxisome proliferator‐activated receptor‐gamma activation in human non small‐cell lung cancer is mediated via proline oxidase‐induced reactive oxygen species formation. Mol. Pharmacol. 72:674–685. 17535976 10.1124/mol.107.035584

[33] Tamma, S. M. , B. Shorter , K. L. Toh , R. Moldwin , and B. Gordon . 2015 Influence of polyunsaturated fatty acids on urologic inflammation. Int. Urol. Nephrol. 47:1753–1761. 26411429 10.1007/s11255-015-1108-8

[34] Gecit, I. , M. Aslan , M. Gunes , N. Pirincci , R. Esen , H. Demir , et al. 2012 Serum prolidase activity, oxidative stress, and nitric oxide levels in patients with bladder cancer. J. Cancer Res. Clin. Oncol. 138:739–743. 22258852 10.1007/s00432-011-1136-4 PMC3325420

[35] Surjana, D. , G. M. Halliday , and D. L. Damian . 2010 Role of nicotinamide in DNA damage, mutagenesis, and DNA repair. J. Nucleic. Acids 2010:157591, 13 pages. 20725615 10.4061/2010/157591 PMC2915624

[36] Chau, L. Y. 2015 Heme oxygenase‐1: emerging target of cancer therapy. J. Biomed. Sci. 22:22. 25885228 10.1186/s12929-015-0128-0 PMC4380252

[37] Sucher, R. , K. Kurz , G. Weiss , R. Margreiter , D. Fuchs , and G. Brandacher . 2010 IDO‐Mediated Tryptophan Degradation in the Pathogenesis of Malignant Tumor Disease. Int. J. Tryptophan Res. 3:113–120. 22084593 10.4137/ijtr.s4157 PMC3195236

